# Crystal structure of {4-[10,15,20-tris­(4-meth­oxy­phen­yl)porphyrin-5-yl]benzyl 2-diazo­acetato}­zinc(II)

**DOI:** 10.1107/S2056989020001085

**Published:** 2020-01-31

**Authors:** Daniel Carrie, Thierry Roisnel, Gerard Simonneaux

**Affiliations:** a Univ Rennes, CNRS, ISCR-UMR6226, F-35000 Rennes, France

**Keywords:** crystal structure, diazo ester, porphyrin, zinc

## Abstract

In the title compound, [Zn(C_50_H_36_N_6_O_5_)], the Zn^II^ cation is chelated by four pyrrole N atoms of the porphyrinate anion and coordinated by a symmetry-generated keto O atom of the diazo­ester group in a distorted square-pyramidal geometry. The mean Zn—N(pyrrole) bond length is 2.058 Å and the Zn—O(diazo­ester) bond length is 2.179 (4) Å. The zinc cation is displaced by 0.2202 (13) Å from the N_4_C_20_ mean plane of the porphyrinate anion toward the O atom; the involvement of this atom leads to a [100] polymeric chain in the crystal.

## Abstract 2

The structure of a zinc porphyrin bearing a diazo ester group at the periphery of the porphyrin ring leads to a coordination polymer featuring ZnN_4_O square pyramids.

## Chemical context   

Among various functional groups, diazo derivatives are particularly attractive because of their high reactivities (Ye & McKervey, 1994 ▸). Since porphyrin macrocycles are important fluorescent probes, their functionalization by a diazo group may have many chemical and biological applications (Mix *et al.*, 2016 ▸). However, the present study of the title compound seems to be the only reported X-ray structure of a porphyrin bearing a diazo ester group at the periphery of a porphyrin ring. In contrast, many structures of five-coordinate zinc porphyrins of the type [Zn(Porph)(*L*)] (Porph = is a porphinato ligand and *L* is a neutral ligand) are known in the literature (Nasri *et al.*, 2016 ▸). During the course of our previous studies on diazo compounds (Ferrand *et al.*, 2005 ▸; Galardon *et al.*, 2000 ▸), we reported the use of metalloporphyrins for catalytic cyclo­propanation and the insertion of diazo­ketone compounds in N—H bonds. (Nicolas *et al.*, 2008 ▸, Nicolas *et al.*, 2009 ▸).
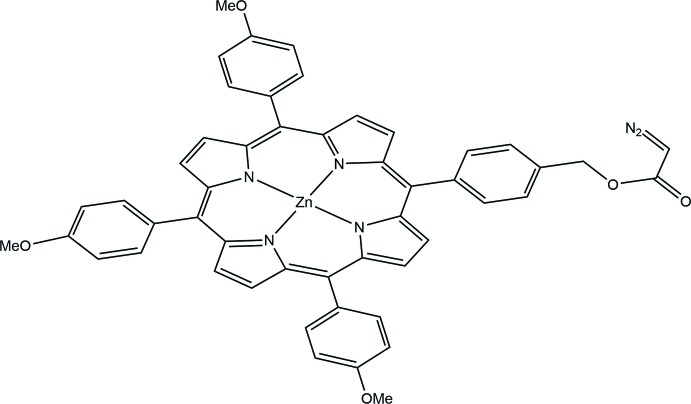


In this work, we describe the crystal structure of the zinc porphyrin title complex, (I), to obtain more insight into the structural relationship of zinc and a diazo­ester group (Carrie *et al.*, 2016 ▸; Fleischer & Shachter, 1991 ▸)

## Structural commentary   

**Figure 1:**
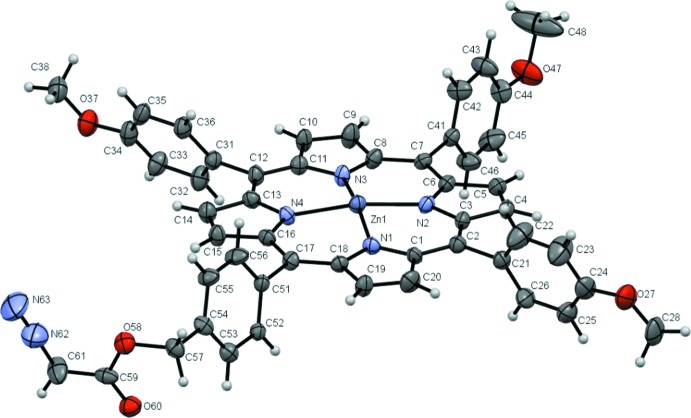
The mol­ecular structure of (I) showing 50% displacement ellipsoids.

**Figure 2:**
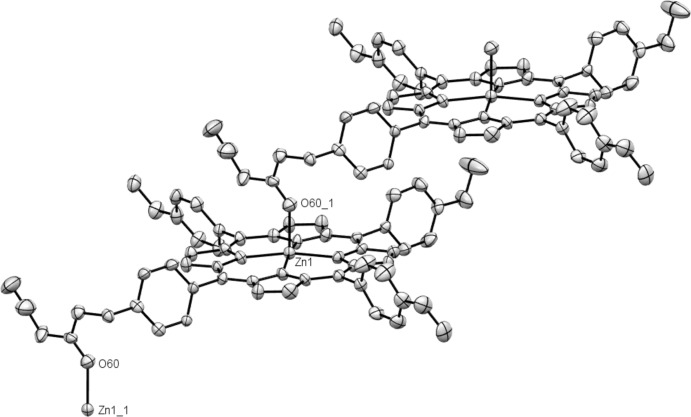
Fragment of the polymeric structure of (I): atoms Zn1_1 and O60_1 are generated by the symmetry operations *x* + 1, *y*, *z* and *x* − 1, *y*, *z*, respectively.

The asymmetric unit of (I) is shown in Fig. 1 ▸. In the crystal, the structure is a one-dimensional polymer, wherein the Zn^2+^ ion bonds to four pyrrole nitrogen atoms and to the diazo ester oxygen atom of an adjacent molecule [O60^i^: symmetry code: (i) *x* − 1, *y*, *z*], thereby defining the propagation of the chain, Figs. 2 ▸ and 3 ▸. The bond lengths from the zinc ion to the pyrrole nitro­gen atoms span the range 2.046 (4)–2.073 (4) Å, which is comparable with those reported in the literature (Nasri *et al.*, 2016 ▸). The Zn—O(diazo­ester) bond length is 2.179 (4) Å, which is slightly shorter than that of ZnTCPP(acetone) (TCPP = meso-tetra­(4-carb­oxy­phen­yl)porphyrin) [2.222 (2) Å; Chen *et al.*, 2014 ▸]. The bond lengths of the diazo group have been previously estimated from X-ray data and *ab initio* calculations for a series of diazo­compounds (average parameters: N—N 1.1189 and N—C = 1.3263 Å). Remarkably little variance in these bond lengths occurs, even with varied functionality around the diazo moiety (Goodman *et al.*, 1994 ▸). For the title compound, the N—N and C—N bond lengths are 1.112 (10) and 1.285 (10) Å, respectively, which are close to those observed for an aromatic diazo­ketone [N—N =1.117 (8); C—N = 1.316 (9) Å; Yanez *et al.*, 2003 ▸].

## Supra­molecular features   

**Figure 3:**
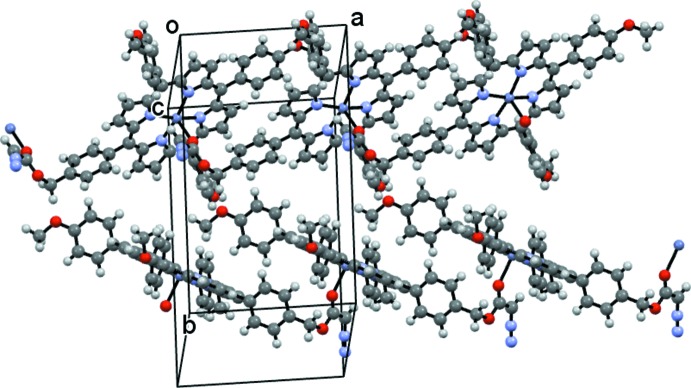
The packing of (I) showing side-by-side polymeric chains propagating in the [100] direction.

Fig. 2 ▸ shows the polymeric nature of (I) consisting of infinite [100] chains of Zn porphyrin units with the diazo­ester of one unit coordinated to the zinc atom of another: the mol­ecules are linked together in such a way as to make two different columns of porphyrin planes (Fig. 3 ▸). There are no other significant inter­molecular contacts present.

## Synthesis and crystallization   

5-[4-(Hy­droxy­meth­yl)phen­yl]-10,15,20-(4-(trimeth­oxy)phen­yl)porphyrin and the zinc starting complex were synthesized using previously reported methods (Carrie *et al.*, 2016 ▸).

To a distilled CH_2_Cl_2_ solution (5 ml) of zinc 5-[4-(hy­droxy­meth­yl)phen­yl]-10,15,20-tri­phenyl­porphyrin (100 mg, 0.12 mmol), 3 eq. of 1,8-diazabicyclo[5.4.0]undec-7-ene (DBU; 0.36 mmol) were first added under argon at 273 K and then 1.5 eq. of bromo acetyl bromide (0.2 mmol). The reaction mixture was stirred for 10 min at room temperature. After cooling the solution again to 273 K, a THF solution of di­tosyl­hydrazine (2 eq.) and DBU (5 eq.) was added, and the mixture was stirred for 30 min at room temperature. The solution was then evaporated, dissolved in CH_2_Cl_2_ and purified through a silica gel column (CH_2_Cl_2_). Yield = 70%. Red prisms of (I) were obtained by diffusion of pentane into a di­chloro­methane solution. UV/VIS (CH_2_Cl_2_): *l*_max_, nm: 421, 548, 592.

## Refinement   

Crystal data, data collection and structure refinement details are summarized in Table 1 ▸. The contribution of the disordered solvents to the calculated structure factors was estimated following the BYPASS algorithm (Sluis & Spek, 1990 ▸), implemented as the SQUEEZE option in *PLATON* (Spek, 2015 ▸). H atoms were finally included in their calculated positions (C—H = 0.95–0.98 Å) and refined as riding atoms with *U*_iso_(H) = 1.2*U*_eq_(C) or 1.5*U*_eq_(methyl C). The methyl H atoms were allowed to rotate, but not to tip, to best fit the electron density. The crystal studied was refined as an inversion twin.

## Supplementary Material

Crystal structure: contains datablock(s) I. DOI: 10.1107/S2056989020001085/hb7871sup1.cif

Structure factors: contains datablock(s) I. DOI: 10.1107/S2056989020001085/hb7871Isup2.hkl

CCDC reference: 1968288

Additional supporting information:  crystallographic information; 3D view; checkCIF report

## Figures and Tables

**Table 1:** Experimental details

Crystal data	
Chemical formula	[Zn(C_50_H_36_N_6_O_5_)]
*M*_r_	866.22
Crystal system, space group	Monoclinic, *P*2_1_
Temperature (K)	150
*a*, *b*, *c* (Å)	10.8054 (8), 19.3938 (14), 12.7467 (10)
β (°)	98.523 (3)
*V* (Å^3^)	2641.7 (3)
*Z*	2
Radiation type	Mo *K*α
μ (mm^−1^)	0.51
Crystal size (mm)	0.32 × 0.30 × 0.10

Data collection	
Diffractometer	D8 VENTURE Bruker AXS
Absorption correction	Multi-scan (*SADABS*; Bruker, 2014 ▸)
*T*_min_, *T*_max_	0.704, 0.950
No. of measured, independent and observed [*I* > 2σ(*I*)] reflections	28170, 12068, 9931
*R*_int_	0.047
(sin θ/λ)_max_ (Å^−1^)	0.650

Refinement	
*R*[*F* ^2^ > 2σ(*F* ^2^)], *wR*(*F* ^2^), *S*	0.051, 0.139, 1.04
No. of reflections	12068
No. of parameters	563
No. of restraints	1
H-atom treatment	H-atom parameters constrained
Δρ_max_, Δρ_min_ (e Å^−3^)	0.43, −0.42
Absolute structure	Flack (1983 ▸)
Absolute structure parameter	0.033 (15)

Computer programs: *SAINT* (Bruker, 2014 ▸), *APEX3* (Bruker, 2015 ▸), *SIR97* (Altomare *et al.*, 1999 ▸), *SHELXL2018/3* (Sheldrick, 2015 ▸), *SXGRAPH* (Farrugia, 1999 ▸), *Mercury* (Macrae *et al.*, 2008 ▸) and *CRYSCALC* (Roisnel, local program, 2019).

## supplementary crystallographic information

### Crystal data

**Table:**

[Zn(C_50_H_36_N_6_O_5_)]	*F*(000) = 896
*M**_r_* = 866.22	*D*_x_ = 1.089 Mg m^−^^3^
Monoclinic, *P*2_1_	Mo *K*α radiation, λ = 0.71073 Å
*a* = 10.8054 (8) Å	Cell parameters from 9886 reflections
*b* = 19.3938 (14) Å	θ = 2.5–27.4°
*c* = 12.7467 (10) Å	µ = 0.51 mm^−^^1^
β = 98.523 (3)°	*T* = 150 K
*V* = 2641.7 (3) Å^3^	Prism, red
*Z* = 2	0.32 × 0.30 × 0.10 mm

### Data collection

**Table:**

D8 VENTURE Bruker AXS diffractometer	12068 independent reflections
Radiation source: Incoatec microfocus sealed tube	9931 reflections with *I* > 2σ(*I*)
Multilayer monochromator	*R*_int_ = 0.047
Detector resolution: 10.4167 pixels mm^-1^	θ_max_ = 27.5°, θ_min_ = 2.1°
rotation images scans	*h* = −14→13
Absorption correction: multi-scan (SADABS; Bruker, 2014)	*k* = −24→25
*T*_min_ = 0.704, *T*_max_ = 0.950	*l* = −16→16
28170 measured reflections	

### Refinement

**Table:**

Refinement on *F*^2^	Secondary atom site location: difference Fourier map
Least-squares matrix: full	Hydrogen site location: inferred from neighbouring sites
*R*[*F*^2^ > 2σ(*F*^2^)] = 0.051	H-atom parameters constrained
*wR*(*F*^2^) = 0.139	*w* = 1/[σ^2^(*F*_o_^2^) + (0.0738*P*)^2^ + 1.1085*P*] where *P* = (*F*_o_^2^ + 2*F*_c_^2^)/3
*S* = 1.04	(Δ/σ)_max_ = 0.024
12068 reflections	Δρ_max_ = 0.43 e Å^−^^3^
563 parameters	Δρ_min_ = −0.42 e Å^−^^3^
1 restraint	Absolute structure: Flack (1983)
Primary atom site location: structure-invariant direct methods	Absolute structure parameter: 0.033 (15)

### Special details

**Table:**

Geometry. All esds (except the esd in the dihedral angle between two l.s. planes) are estimated using the full covariance matrix. The cell esds are taken into account individually in the estimation of esds in distances, angles and torsion angles; correlations between esds in cell parameters are only used when they are defined by crystal symmetry. An approximate (isotropic) treatment of cell esds is used for estimating esds involving l.s. planes.
Refinement. Refinement of F^2^ against ALL reflections. The weighted R-factor wR and goodness of fit S are based on F^2^, conventional R-factors R are based on F, with F set to zero for negative F^2^. The threshold expression of F^2^ > 2sigma(F^2^) is used only for calculating R-factors(gt) etc. and is not relevant to the choice of reflections for refinement. R-factors based on F^2^ are statistically about twice as large as those based on F, and R- factors based on ALL data will be even larger.

### Fractional atomic coordinates and isotropic or equivalent isotropic displacement parameters (Å^2^)

**Table:**

	*x*	*y*	*z*	*U*_iso_*/*U*_eq_	
Zn1	0.00556 (5)	0.70060 (3)	0.62458 (4)	0.02718 (14)	
N1	0.0900 (4)	0.7458 (2)	0.5081 (3)	0.0273 (9)	
N2	−0.1436 (4)	0.6737 (2)	0.5139 (3)	0.0279 (9)	
N3	−0.0613 (4)	0.6355 (2)	0.7323 (3)	0.0280 (9)	
N4	0.1702 (3)	0.7083 (3)	0.7288 (3)	0.0264 (8)	
C1	0.0353 (4)	0.7599 (3)	0.4074 (4)	0.0258 (10)	
C2	−0.0846 (5)	0.7376 (3)	0.3602 (4)	0.0284 (10)	
C3	−0.1656 (4)	0.6983 (3)	0.4102 (3)	0.0282 (9)	
C4	−0.2887 (5)	0.6744 (3)	0.3615 (4)	0.0294 (10)	
H4	−0.327042	0.683287	0.290868	0.035*	
C5	−0.3383 (5)	0.6375 (3)	0.4341 (4)	0.0341 (12)	
H5	−0.417616	0.615398	0.425012	0.041*	
C6	−0.2463 (5)	0.6384 (3)	0.5294 (4)	0.0267 (10)	
C7	−0.2652 (5)	0.6058 (2)	0.6254 (4)	0.0257 (10)	
C8	−0.1758 (5)	0.6049 (3)	0.7185 (4)	0.0304 (11)	
C9	−0.1968 (6)	0.5711 (3)	0.8157 (4)	0.0401 (13)	
H9	−0.269308	0.546393	0.827774	0.048*	
C10	−0.0913 (5)	0.5821 (3)	0.8860 (4)	0.0331 (12)	
H10	−0.075922	0.565878	0.957105	0.040*	
C11	−0.0068 (5)	0.6227 (3)	0.8337 (4)	0.0281 (10)	
C12	0.1111 (5)	0.6462 (3)	0.8823 (4)	0.0309 (11)	
C13	0.1928 (5)	0.6856 (2)	0.8307 (4)	0.0281 (11)	
C14	0.3165 (5)	0.7062 (3)	0.8786 (4)	0.0345 (10)	
H14	0.355695	0.696032	0.948623	0.041*	
C15	0.3662 (5)	0.7428 (3)	0.8045 (4)	0.0327 (11)	
H15	0.446606	0.763638	0.813239	0.039*	
C16	0.2751 (4)	0.7442 (3)	0.7105 (4)	0.0266 (10)	
C17	0.2948 (5)	0.7755 (2)	0.6138 (4)	0.0262 (10)	
C18	0.2070 (5)	0.7756 (2)	0.5209 (4)	0.0263 (10)	
C19	0.2248 (5)	0.8106 (3)	0.4230 (4)	0.0325 (11)	
H19	0.296284	0.836265	0.410923	0.039*	
C20	0.1203 (6)	0.7995 (3)	0.3528 (4)	0.0408 (13)	
H20	0.105179	0.814796	0.281241	0.049*	
C21	−0.1269 (5)	0.7562 (3)	0.2450 (4)	0.0305 (11)	
C22	−0.1727 (9)	0.8212 (4)	0.2161 (5)	0.061 (2)	
H22	−0.177863	0.854917	0.269352	0.073*	
C23	−0.2109 (9)	0.8379 (4)	0.1107 (6)	0.067 (2)	
H23	−0.236839	0.883653	0.092358	0.080*	
C24	−0.2119 (6)	0.7893 (3)	0.0320 (4)	0.0434 (14)	
C25	−0.1649 (6)	0.7242 (3)	0.0607 (5)	0.0475 (16)	
H25	−0.162430	0.689600	0.008378	0.057*	
C26	−0.1217 (5)	0.7103 (3)	0.1663 (4)	0.0395 (13)	
H26	−0.086674	0.666223	0.184301	0.047*	
O27	−0.2603 (5)	0.8092 (3)	−0.0664 (3)	0.0585 (13)	
C28	−0.2763 (7)	0.7571 (4)	−0.1445 (5)	0.0555 (18)	
H28A	−0.320784	0.717971	−0.118881	0.083*	
H28B	−0.194202	0.741805	−0.159425	0.083*	
H28C	−0.324992	0.775327	−0.209602	0.083*	
C31	0.1521 (5)	0.6269 (3)	0.9941 (4)	0.0298 (10)	
C32	0.1745 (6)	0.5584 (3)	1.0261 (5)	0.0419 (13)	
H32	0.160518	0.522090	0.975640	0.050*	
C33	0.2178 (6)	0.5436 (3)	1.1330 (4)	0.0380 (12)	
H33	0.231397	0.496819	1.153829	0.046*	
C34	0.2405 (6)	0.5930 (3)	1.2067 (5)	0.0424 (14)	
C35	0.2166 (6)	0.6622 (3)	1.1777 (4)	0.0397 (13)	
H35	0.229459	0.697573	1.229692	0.048*	
C36	0.1744 (5)	0.6783 (3)	1.0732 (4)	0.0363 (12)	
H36	0.159940	0.725241	1.053906	0.044*	
O37	0.2872 (4)	0.5733 (2)	1.3082 (3)	0.0470 (10)	
C38	0.3071 (7)	0.6249 (3)	1.3886 (5)	0.0478 (15)	
H38A	0.363013	0.660543	1.367831	0.072*	
H38B	0.345083	0.603894	1.455623	0.072*	
H38C	0.226739	0.645802	1.397698	0.072*	
C41	−0.3889 (5)	0.5724 (2)	0.6304 (4)	0.0260 (10)	
C42	−0.4812 (6)	0.6063 (3)	0.6729 (5)	0.0387 (13)	
H42	−0.465968	0.652103	0.697849	0.046*	
C43	−0.5957 (6)	0.5764 (3)	0.6808 (6)	0.0464 (15)	
H43	−0.658937	0.602078	0.707695	0.056*	
C44	−0.6171 (5)	0.5084 (3)	0.6488 (5)	0.0353 (12)	
C45	−0.5207 (7)	0.4725 (3)	0.6066 (5)	0.0497 (16)	
H45	−0.532431	0.425747	0.585217	0.060*	
C46	−0.4128 (6)	0.5049 (3)	0.5969 (6)	0.0454 (15)	
H46	−0.350744	0.480710	0.566033	0.054*	
O47	−0.7270 (4)	0.4728 (2)	0.6512 (5)	0.0628 (14)	
C48	−0.8253 (10)	0.5098 (5)	0.6903 (13)	0.123 (5)	
H48A	−0.827708	0.557236	0.663782	0.184*	
H48B	−0.809986	0.510177	0.768063	0.184*	
H48C	−0.905543	0.487177	0.665944	0.184*	
C54	0.6527 (5)	0.8721 (3)	0.6153 (4)	0.0297 (11)	
C55	0.5618 (5)	0.9019 (3)	0.6678 (5)	0.0357 (12)	
H55	0.579464	0.944167	0.704745	0.043*	
C56	0.4463 (6)	0.8711 (3)	0.6671 (5)	0.0385 (13)	
H56	0.386237	0.892012	0.704379	0.046*	
C51	0.4173 (5)	0.8096 (3)	0.6121 (4)	0.0271 (10)	
C52	0.5070 (5)	0.7809 (2)	0.5572 (4)	0.0256 (10)	
H52	0.488255	0.739651	0.517825	0.031*	
C53	0.6239 (5)	0.8117 (3)	0.5589 (4)	0.0306 (11)	
H53	0.684042	0.791255	0.521289	0.037*	
O58	0.8581 (4)	0.89908 (19)	0.7153 (3)	0.0390 (9)	
C59	0.9262 (5)	0.8416 (3)	0.7264 (5)	0.0342 (12)	
C57	0.7751 (5)	0.9070 (3)	0.6121 (4)	0.0326 (11)	
H57A	0.816041	0.886703	0.554810	0.039*	
H57B	0.760818	0.956611	0.596421	0.039*	
O60	0.9171 (4)	0.7974 (2)	0.6583 (3)	0.0410 (9)	
C61	1.0062 (7)	0.8346 (3)	0.8270 (6)	0.0530 (18)	
H61	1.059875	0.795791	0.839911	0.064*	
N62	1.0053 (7)	0.8808 (4)	0.8992 (6)	0.076 (2)	
N63	1.0095 (9)	0.9222 (5)	0.9599 (7)	0.109 (4)	

### Atomic displacement parameters (Å^2^)

**Table:**

	*U*^11^	*U*^22^	*U*^33^	*U*^12^	*U*^13^	*U*^23^
Zn1	0.0255 (2)	0.0322 (3)	0.0238 (2)	−0.0034 (3)	0.00351 (18)	0.0022 (3)
N1	0.022 (2)	0.033 (2)	0.027 (2)	−0.0035 (17)	0.0065 (17)	0.0021 (17)
N2	0.031 (2)	0.033 (2)	0.019 (2)	−0.0007 (17)	0.0022 (17)	−0.0004 (16)
N3	0.027 (2)	0.031 (2)	0.024 (2)	−0.0103 (17)	0.0004 (17)	0.0036 (17)
N4	0.0224 (17)	0.031 (2)	0.0257 (18)	0.0005 (19)	0.0030 (14)	0.0083 (19)
C1	0.020 (2)	0.036 (3)	0.022 (2)	0.0022 (19)	0.0038 (18)	0.0054 (19)
C2	0.028 (2)	0.032 (2)	0.025 (2)	0.000 (2)	0.0030 (19)	0.001 (2)
C3	0.029 (2)	0.034 (2)	0.020 (2)	0.004 (3)	−0.0003 (16)	0.000 (2)
C4	0.025 (2)	0.039 (2)	0.023 (2)	−0.004 (2)	−0.0003 (19)	−0.001 (2)
C5	0.029 (3)	0.045 (3)	0.028 (3)	−0.001 (2)	0.005 (2)	−0.005 (2)
C6	0.029 (3)	0.031 (2)	0.021 (2)	0.000 (2)	0.0030 (19)	−0.0022 (18)
C7	0.029 (3)	0.027 (2)	0.023 (2)	−0.0018 (19)	0.0078 (19)	−0.0022 (18)
C8	0.036 (3)	0.034 (3)	0.022 (2)	−0.006 (2)	0.004 (2)	0.0011 (19)
C9	0.038 (3)	0.055 (3)	0.027 (3)	−0.016 (3)	0.002 (2)	0.008 (2)
C10	0.028 (3)	0.048 (3)	0.023 (3)	−0.011 (2)	0.001 (2)	0.010 (2)
C11	0.028 (3)	0.030 (2)	0.026 (2)	−0.005 (2)	0.004 (2)	0.0047 (19)
C12	0.029 (3)	0.038 (3)	0.024 (2)	0.001 (2)	−0.002 (2)	0.006 (2)
C13	0.025 (2)	0.032 (3)	0.028 (2)	−0.0006 (18)	0.0061 (19)	0.0038 (17)
C14	0.031 (2)	0.042 (3)	0.030 (2)	−0.004 (3)	0.0008 (18)	0.004 (3)
C15	0.026 (3)	0.042 (3)	0.029 (3)	−0.008 (2)	0.001 (2)	0.004 (2)
C16	0.020 (2)	0.032 (2)	0.029 (2)	−0.0027 (19)	0.0053 (19)	0.0004 (19)
C17	0.026 (2)	0.024 (2)	0.029 (3)	−0.0067 (18)	0.003 (2)	−0.0015 (18)
C18	0.026 (2)	0.031 (2)	0.022 (2)	−0.0014 (19)	0.0065 (19)	0.0029 (18)
C19	0.031 (3)	0.041 (3)	0.025 (3)	−0.010 (2)	0.003 (2)	0.005 (2)
C20	0.049 (3)	0.051 (3)	0.023 (3)	0.002 (3)	0.008 (2)	0.012 (2)
C21	0.028 (3)	0.039 (3)	0.025 (3)	0.000 (2)	0.005 (2)	0.006 (2)
C22	0.103 (6)	0.042 (3)	0.036 (3)	0.013 (4)	0.006 (4)	0.006 (3)
C23	0.102 (7)	0.043 (4)	0.049 (4)	0.018 (4)	−0.006 (4)	0.005 (3)
C24	0.046 (3)	0.060 (4)	0.025 (3)	0.005 (3)	0.010 (2)	0.019 (3)
C25	0.051 (4)	0.062 (4)	0.029 (3)	0.014 (3)	0.006 (3)	0.001 (2)
C26	0.044 (3)	0.038 (3)	0.035 (3)	0.017 (3)	−0.001 (2)	0.004 (2)
O27	0.065 (3)	0.079 (3)	0.029 (2)	0.018 (3)	0.002 (2)	0.012 (2)
C28	0.058 (4)	0.080 (5)	0.025 (3)	−0.002 (4)	−0.004 (3)	0.017 (3)
C31	0.028 (3)	0.034 (2)	0.027 (3)	−0.004 (2)	0.002 (2)	−0.001 (2)
C32	0.049 (3)	0.041 (3)	0.032 (3)	−0.002 (3)	−0.003 (3)	0.008 (2)
C33	0.052 (3)	0.033 (3)	0.029 (3)	−0.005 (2)	0.002 (2)	0.007 (2)
C34	0.040 (3)	0.054 (4)	0.030 (3)	−0.007 (3)	−0.003 (2)	0.009 (3)
C35	0.039 (3)	0.055 (3)	0.024 (3)	−0.008 (3)	0.001 (2)	−0.003 (2)
C36	0.037 (3)	0.042 (3)	0.029 (3)	0.001 (2)	0.000 (2)	0.006 (2)
O37	0.057 (3)	0.046 (2)	0.035 (2)	0.000 (2)	−0.002 (2)	0.0109 (18)
C38	0.048 (4)	0.048 (3)	0.043 (4)	−0.002 (3)	−0.009 (3)	−0.002 (3)
C41	0.030 (3)	0.027 (2)	0.021 (2)	−0.004 (2)	0.0042 (19)	−0.0008 (18)
C42	0.041 (3)	0.029 (3)	0.050 (4)	−0.008 (2)	0.017 (3)	−0.004 (2)
C43	0.032 (3)	0.039 (3)	0.072 (5)	−0.004 (2)	0.018 (3)	−0.004 (3)
C44	0.034 (3)	0.026 (2)	0.045 (3)	−0.013 (2)	0.005 (2)	0.004 (2)
C45	0.059 (4)	0.037 (3)	0.056 (4)	−0.017 (3)	0.018 (3)	−0.016 (3)
C46	0.037 (3)	0.042 (3)	0.059 (4)	−0.005 (3)	0.015 (3)	−0.019 (3)
O47	0.038 (2)	0.047 (2)	0.108 (4)	−0.015 (2)	0.025 (3)	−0.004 (3)
C48	0.079 (7)	0.052 (5)	0.259 (17)	−0.015 (5)	0.096 (9)	−0.032 (7)
C54	0.030 (3)	0.023 (2)	0.036 (3)	0.003 (2)	0.006 (2)	0.008 (2)
C55	0.036 (3)	0.033 (3)	0.039 (3)	−0.006 (2)	0.011 (2)	−0.013 (2)
C56	0.043 (3)	0.031 (3)	0.044 (3)	−0.003 (2)	0.015 (3)	−0.014 (2)
C51	0.024 (2)	0.034 (2)	0.022 (2)	−0.002 (2)	0.0009 (19)	−0.0019 (19)
C52	0.024 (2)	0.028 (2)	0.024 (2)	−0.0025 (19)	0.0008 (19)	−0.0022 (18)
C53	0.031 (3)	0.030 (2)	0.031 (3)	0.001 (2)	0.006 (2)	0.007 (2)
O58	0.041 (2)	0.0315 (19)	0.043 (2)	−0.0015 (16)	0.0017 (18)	−0.0020 (16)
C59	0.028 (3)	0.027 (2)	0.050 (3)	−0.003 (2)	0.015 (2)	−0.007 (2)
C57	0.026 (3)	0.032 (2)	0.040 (3)	−0.004 (2)	0.005 (2)	0.001 (2)
O60	0.044 (2)	0.037 (2)	0.042 (2)	0.0002 (17)	0.0075 (18)	−0.0055 (17)
C61	0.055 (4)	0.033 (3)	0.063 (4)	0.002 (3)	−0.017 (3)	−0.014 (3)
N62	0.079 (5)	0.080 (5)	0.066 (4)	0.030 (4)	0.001 (4)	−0.014 (4)
N63	0.109 (7)	0.118 (7)	0.089 (6)	0.034 (6)	−0.021 (5)	−0.066 (6)

### Geometric parameters (Å, º)

**Table:**

Zn1—N2	2.046 (4)	C26—H26	0.9500
Zn1—N1	2.051 (4)	O27—C28	1.411 (9)
Zn1—N4	2.062 (4)	C28—H28A	0.9800
Zn1—N3	2.073 (4)	C28—H28B	0.9800
Zn1—O60^i^	2.179 (4)	C28—H28C	0.9800
N1—C1	1.359 (6)	C31—C32	1.401 (8)
N1—C18	1.378 (6)	C31—C36	1.413 (8)
N2—C6	1.343 (7)	C32—C33	1.403 (8)
N2—C3	1.392 (6)	C32—H32	0.9500
N3—C8	1.359 (7)	C33—C34	1.340 (8)
N3—C11	1.360 (7)	C33—H33	0.9500
N4—C13	1.359 (6)	C34—O37	1.371 (7)
N4—C16	1.379 (6)	C34—C35	1.405 (9)
C1—C2	1.413 (7)	C35—C36	1.378 (8)
C1—C20	1.452 (7)	C35—H35	0.9500
C2—C3	1.385 (7)	C36—H36	0.9500
C2—C21	1.517 (7)	O37—C38	1.426 (8)
C3—C4	1.457 (7)	C38—H38A	0.9800
C4—C5	1.342 (8)	C38—H38B	0.9800
C4—H4	0.9500	C38—H38C	0.9800
C5—C6	1.451 (7)	C41—C42	1.372 (8)
C5—H5	0.9500	C41—C46	1.389 (8)
C6—C7	1.419 (7)	C42—C43	1.383 (8)
C7—C8	1.415 (7)	C42—H42	0.9500
C7—C41	1.495 (7)	C43—C44	1.390 (8)
C8—C9	1.449 (7)	C43—H43	0.9500
C9—C10	1.358 (8)	C44—O47	1.377 (7)
C9—H9	0.9500	C44—C45	1.423 (9)
C10—C11	1.443 (7)	C45—C46	1.345 (9)
C10—H10	0.9500	C45—H45	0.9500
C11—C12	1.407 (7)	C46—H46	0.9500
C12—C13	1.403 (7)	O47—C48	1.431 (10)
C12—C31	1.476 (7)	C48—H48A	0.9800
C13—C14	1.441 (7)	C48—H48B	0.9800
C14—C15	1.354 (7)	C48—H48C	0.9800
C14—H14	0.9500	C54—C53	1.384 (7)
C15—C16	1.434 (7)	C54—C55	1.394 (8)
C15—H15	0.9500	C54—C57	1.492 (7)
C16—C17	1.418 (7)	C55—C56	1.381 (8)
C17—C18	1.404 (7)	C55—H55	0.9500
C17—C51	1.483 (7)	C56—C51	1.397 (7)
C18—C19	1.458 (7)	C56—H56	0.9500
C19—C20	1.350 (8)	C51—C52	1.393 (7)
C19—H19	0.9500	C52—C53	1.395 (7)
C20—H20	0.9500	C52—H52	0.9500
C21—C26	1.347 (8)	C53—H53	0.9500
C21—C22	1.384 (8)	O58—C59	1.332 (6)
C22—C23	1.384 (10)	O58—C57	1.486 (7)
C22—H22	0.9500	C59—O60	1.213 (6)
C23—C24	1.374 (10)	C59—C61	1.443 (9)
C23—H23	0.9500	C57—H57A	0.9900
C24—O27	1.342 (7)	C57—H57B	0.9900
C24—C25	1.389 (9)	C61—N62	1.285 (10)
C25—C26	1.385 (8)	C61—H61	0.9500
C25—H25	0.9500	N62—N63	1.112 (10)
			
N2—Zn1—N1	89.95 (17)	C26—C25—H25	120.3
N2—Zn1—N4	168.25 (18)	C24—C25—H25	120.3
N1—Zn1—N4	90.10 (16)	C21—C26—C25	123.3 (5)
N2—Zn1—N3	89.15 (16)	C21—C26—H26	118.3
N1—Zn1—N3	167.43 (17)	C25—C26—H26	118.3
N4—Zn1—N3	88.25 (16)	C24—O27—C28	116.4 (5)
N2—Zn1—O60^i^	91.84 (16)	O27—C28—H28A	109.5
N1—Zn1—O60^i^	91.71 (16)	O27—C28—H28B	109.5
N4—Zn1—O60^i^	99.90 (17)	H28A—C28—H28B	109.5
N3—Zn1—O60^i^	100.85 (17)	O27—C28—H28C	109.5
C1—N1—C18	106.9 (4)	H28A—C28—H28C	109.5
C1—N1—Zn1	126.3 (3)	H28B—C28—H28C	109.5
C18—N1—Zn1	126.3 (3)	C32—C31—C36	117.2 (5)
C6—N2—C3	106.6 (4)	C32—C31—C12	122.4 (5)
C6—N2—Zn1	127.7 (3)	C36—C31—C12	120.4 (5)
C3—N2—Zn1	125.0 (3)	C31—C32—C33	119.7 (5)
C8—N3—C11	107.5 (4)	C31—C32—H32	120.2
C8—N3—Zn1	125.4 (3)	C33—C32—H32	120.2
C11—N3—Zn1	126.5 (3)	C34—C33—C32	122.3 (5)
C13—N4—C16	106.8 (4)	C34—C33—H33	118.8
C13—N4—Zn1	127.5 (3)	C32—C33—H33	118.8
C16—N4—Zn1	125.5 (3)	C33—C34—O37	117.8 (5)
N1—C1—C2	125.7 (4)	C33—C34—C35	119.4 (5)
N1—C1—C20	110.0 (4)	O37—C34—C35	122.8 (6)
C2—C1—C20	124.3 (5)	C36—C35—C34	119.5 (5)
C3—C2—C1	125.1 (5)	C36—C35—H35	120.2
C3—C2—C21	117.1 (4)	C34—C35—H35	120.2
C1—C2—C21	117.7 (4)	C35—C36—C31	121.8 (5)
C2—C3—N2	126.5 (4)	C35—C36—H36	119.1
C2—C3—C4	125.4 (4)	C31—C36—H36	119.1
N2—C3—C4	108.2 (4)	C34—O37—C38	118.5 (5)
C5—C4—C3	108.0 (5)	O37—C38—H38A	109.5
C5—C4—H4	126.0	O37—C38—H38B	109.5
C3—C4—H4	126.0	H38A—C38—H38B	109.5
C4—C5—C6	106.1 (5)	O37—C38—H38C	109.5
C4—C5—H5	127.0	H38A—C38—H38C	109.5
C6—C5—H5	127.0	H38B—C38—H38C	109.5
N2—C6—C7	125.6 (4)	C42—C41—C46	117.4 (5)
N2—C6—C5	111.2 (4)	C42—C41—C7	121.1 (4)
C7—C6—C5	123.3 (5)	C46—C41—C7	121.5 (5)
C8—C7—C6	124.2 (5)	C41—C42—C43	122.6 (5)
C8—C7—C41	117.6 (4)	C41—C42—H42	118.7
C6—C7—C41	118.2 (4)	C43—C42—H42	118.7
N3—C8—C7	126.8 (4)	C42—C43—C44	119.2 (6)
N3—C8—C9	110.0 (5)	C42—C43—H43	120.4
C7—C8—C9	123.3 (5)	C44—C43—H43	120.4
C10—C9—C8	105.7 (5)	O47—C44—C43	125.1 (6)
C10—C9—H9	127.2	O47—C44—C45	116.5 (5)
C8—C9—H9	127.2	C43—C44—C45	118.4 (5)
C9—C10—C11	107.9 (5)	C46—C45—C44	120.0 (5)
C9—C10—H10	126.1	C46—C45—H45	120.0
C11—C10—H10	126.1	C44—C45—H45	120.0
N3—C11—C12	126.6 (4)	C45—C46—C41	122.4 (6)
N3—C11—C10	108.9 (4)	C45—C46—H46	118.8
C12—C11—C10	124.5 (5)	C41—C46—H46	118.8
C13—C12—C11	124.2 (5)	C44—O47—C48	116.4 (6)
C13—C12—C31	118.1 (5)	O47—C48—H48A	109.5
C11—C12—C31	117.8 (5)	O47—C48—H48B	109.5
N4—C13—C12	126.0 (5)	H48A—C48—H48B	109.5
N4—C13—C14	109.8 (4)	O47—C48—H48C	109.5
C12—C13—C14	124.2 (5)	H48A—C48—H48C	109.5
C15—C14—C13	106.8 (4)	H48B—C48—H48C	109.5
C15—C14—H14	126.6	C53—C54—C55	118.7 (5)
C13—C14—H14	126.6	C53—C54—C57	120.1 (5)
C14—C15—C16	107.4 (5)	C55—C54—C57	121.0 (5)
C14—C15—H15	126.3	C56—C55—C54	121.2 (5)
C16—C15—H15	126.3	C56—C55—H55	119.4
N4—C16—C17	126.6 (4)	C54—C55—H55	119.4
N4—C16—C15	109.2 (4)	C55—C56—C51	120.5 (5)
C17—C16—C15	124.2 (4)	C55—C56—H56	119.7
C18—C17—C16	124.4 (4)	C51—C56—H56	119.7
C18—C17—C51	118.7 (4)	C52—C51—C56	118.2 (5)
C16—C17—C51	117.0 (4)	C52—C51—C17	121.3 (4)
N1—C18—C17	126.4 (4)	C56—C51—C17	120.5 (5)
N1—C18—C19	109.2 (4)	C51—C52—C53	121.1 (5)
C17—C18—C19	124.4 (5)	C51—C52—H52	119.4
C20—C19—C18	106.8 (5)	C53—C52—H52	119.4
C20—C19—H19	126.6	C54—C53—C52	120.3 (5)
C18—C19—H19	126.6	C54—C53—H53	119.9
C19—C20—C1	107.0 (5)	C52—C53—H53	119.9
C19—C20—H20	126.5	C59—O58—C57	115.6 (4)
C1—C20—H20	126.5	O60—C59—O58	122.0 (5)
C26—C21—C22	117.1 (5)	O60—C59—C61	123.0 (5)
C26—C21—C2	121.6 (5)	O58—C59—C61	114.9 (5)
C22—C21—C2	121.3 (5)	O58—C57—C54	110.5 (4)
C23—C22—C21	120.9 (6)	O58—C57—H57A	109.6
C23—C22—H22	119.5	C54—C57—H57A	109.6
C21—C22—H22	119.5	O58—C57—H57B	109.6
C24—C23—C22	121.2 (6)	C54—C57—H57B	109.6
C24—C23—H23	119.4	H57A—C57—H57B	108.1
C22—C23—H23	119.4	C59—O60—Zn1^ii^	139.3 (4)
O27—C24—C23	116.5 (6)	N62—C61—C59	120.3 (6)
O27—C24—C25	125.7 (6)	N62—C61—H61	119.9
C23—C24—C25	117.8 (5)	C59—C61—H61	119.9
C26—C25—C24	119.4 (6)	N63—N62—C61	176.5 (11)
			
C18—N1—C1—C2	178.4 (5)	C51—C17—C18—C19	−3.6 (7)
Zn1—N1—C1—C2	−9.3 (7)	N1—C18—C19—C20	−1.7 (6)
C18—N1—C1—C20	0.7 (6)	C17—C18—C19—C20	−179.4 (5)
Zn1—N1—C1—C20	173.0 (4)	C18—C19—C20—C1	2.0 (6)
N1—C1—C2—C3	0.9 (8)	N1—C1—C20—C19	−1.8 (6)
C20—C1—C2—C3	178.3 (5)	C2—C1—C20—C19	−179.5 (5)
N1—C1—C2—C21	−177.4 (5)	C3—C2—C21—C26	−77.8 (7)
C20—C1—C2—C21	0.0 (8)	C1—C2—C21—C26	100.6 (6)
C1—C2—C3—N2	−0.6 (9)	C3—C2—C21—C22	102.3 (7)
C21—C2—C3—N2	177.7 (5)	C1—C2—C21—C22	−79.3 (8)
C1—C2—C3—C4	−179.2 (5)	C26—C21—C22—C23	−0.1 (12)
C21—C2—C3—C4	−0.9 (8)	C2—C21—C22—C23	179.9 (7)
C6—N2—C3—C2	179.9 (5)	C21—C22—C23—C24	4.0 (14)
Zn1—N2—C3—C2	8.7 (8)	C22—C23—C24—O27	174.6 (8)
C6—N2—C3—C4	−1.2 (6)	C22—C23—C24—C25	−4.5 (12)
Zn1—N2—C3—C4	−172.5 (3)	O27—C24—C25—C26	−177.7 (6)
C2—C3—C4—C5	179.5 (5)	C23—C24—C25—C26	1.3 (10)
N2—C3—C4—C5	0.6 (6)	C22—C21—C26—C25	−3.2 (10)
C3—C4—C5—C6	0.2 (6)	C2—C21—C26—C25	176.9 (6)
C3—N2—C6—C7	−178.5 (5)	C24—C25—C26—C21	2.6 (10)
Zn1—N2—C6—C7	−7.5 (7)	C23—C24—O27—C28	−172.0 (7)
C3—N2—C6—C5	1.4 (6)	C25—C24—O27—C28	7.0 (10)
Zn1—N2—C6—C5	172.4 (3)	C13—C12—C31—C32	−114.4 (6)
C4—C5—C6—N2	−1.0 (6)	C11—C12—C31—C32	64.5 (7)
C4—C5—C6—C7	178.9 (5)	C13—C12—C31—C36	63.4 (7)
N2—C6—C7—C8	−1.3 (8)	C11—C12—C31—C36	−117.7 (6)
C5—C6—C7—C8	178.8 (5)	C36—C31—C32—C33	−0.2 (8)
N2—C6—C7—C41	176.3 (5)	C12—C31—C32—C33	177.7 (5)
C5—C6—C7—C41	−3.6 (7)	C31—C32—C33—C34	−0.9 (10)
C11—N3—C8—C7	179.2 (5)	C32—C33—C34—O37	−177.5 (6)
Zn1—N3—C8—C7	6.9 (8)	C32—C33—C34—C35	2.2 (10)
C11—N3—C8—C9	0.3 (6)	C33—C34—C35—C36	−2.3 (9)
Zn1—N3—C8—C9	−171.9 (4)	O37—C34—C35—C36	177.4 (6)
C6—C7—C8—N3	1.5 (8)	C34—C35—C36—C31	1.2 (9)
C41—C7—C8—N3	−176.1 (5)	C32—C31—C36—C35	0.1 (8)
C6—C7—C8—C9	−179.8 (5)	C12—C31—C36—C35	−177.9 (5)
C41—C7—C8—C9	2.6 (8)	C33—C34—O37—C38	−177.4 (6)
N3—C8—C9—C10	−0.6 (7)	C35—C34—O37—C38	2.9 (9)
C7—C8—C9—C10	−179.5 (5)	C8—C7—C41—C42	81.1 (7)
C8—C9—C10—C11	0.6 (7)	C6—C7—C41—C42	−96.7 (6)
C8—N3—C11—C12	−178.4 (5)	C8—C7—C41—C46	−95.9 (6)
Zn1—N3—C11—C12	−6.3 (8)	C6—C7—C41—C46	86.3 (7)
C8—N3—C11—C10	0.1 (6)	C46—C41—C42—C43	−1.5 (9)
Zn1—N3—C11—C10	172.2 (4)	C7—C41—C42—C43	−178.6 (6)
C9—C10—C11—N3	−0.5 (7)	C41—C42—C43—C44	2.8 (11)
C9—C10—C11—C12	178.0 (5)	C42—C43—C44—O47	−179.2 (6)
N3—C11—C12—C13	−1.9 (9)	C42—C43—C44—C45	−1.5 (10)
C10—C11—C12—C13	179.9 (5)	O47—C44—C45—C46	176.9 (7)
N3—C11—C12—C31	179.4 (5)	C43—C44—C45—C46	−1.0 (10)
C10—C11—C12—C31	1.1 (8)	C44—C45—C46—C41	2.4 (11)
C16—N4—C13—C12	−179.4 (5)	C42—C41—C46—C45	−1.2 (10)
Zn1—N4—C13—C12	6.5 (8)	C7—C41—C46—C45	175.9 (6)
C16—N4—C13—C14	−1.2 (6)	C43—C44—O47—C48	−0.2 (12)
Zn1—N4—C13—C14	−175.3 (4)	C45—C44—O47—C48	−177.9 (9)
C11—C12—C13—N4	1.8 (8)	C53—C54—C55—C56	2.2 (9)
C31—C12—C13—N4	−179.5 (5)	C57—C54—C55—C56	177.2 (5)
C11—C12—C13—C14	−176.1 (5)	C54—C55—C56—C51	−1.0 (9)
C31—C12—C13—C14	2.6 (8)	C55—C56—C51—C52	−0.8 (9)
N4—C13—C14—C15	1.3 (7)	C55—C56—C51—C17	178.2 (5)
C12—C13—C14—C15	179.5 (5)	C18—C17—C51—C52	−71.5 (6)
C13—C14—C15—C16	−0.9 (6)	C16—C17—C51—C52	108.0 (6)
C13—N4—C16—C17	178.1 (5)	C18—C17—C51—C56	109.5 (6)
Zn1—N4—C16—C17	−7.6 (8)	C16—C17—C51—C56	−71.0 (7)
C13—N4—C16—C15	0.7 (6)	C56—C51—C52—C53	1.5 (8)
Zn1—N4—C16—C15	174.9 (4)	C17—C51—C52—C53	−177.6 (5)
C14—C15—C16—N4	0.2 (6)	C55—C54—C53—C52	−1.5 (8)
C14—C15—C16—C17	−177.4 (5)	C57—C54—C53—C52	−176.6 (5)
N4—C16—C17—C18	1.4 (8)	C51—C52—C53—C54	−0.3 (8)
C15—C16—C17—C18	178.5 (5)	C57—O58—C59—O60	−2.9 (7)
N4—C16—C17—C51	−178.1 (5)	C57—O58—C59—C61	180.0 (5)
C15—C16—C17—C51	−1.0 (7)	C59—O58—C57—C54	86.2 (5)
C1—N1—C18—C17	178.2 (5)	C53—C54—C57—O58	−108.5 (5)
Zn1—N1—C18—C17	6.0 (7)	C55—C54—C57—O58	76.6 (6)
C1—N1—C18—C19	0.5 (6)	O58—C59—O60—Zn1^ii^	174.5 (4)
Zn1—N1—C18—C19	−171.7 (3)	C61—C59—O60—Zn1^ii^	−8.5 (9)
C16—C17—C18—N1	−0.4 (8)	O60—C59—C61—N62	−174.9 (7)
C51—C17—C18—N1	179.1 (5)	O58—C59—C61—N62	2.2 (10)
C16—C17—C18—C19	176.9 (5)		

Symmetry codes: (i) *x*−1, *y*, *z*; (ii) *x*+1, *y*, *z*.
